# Two species–one wavelength detection based on selective optical saturation spectroscopy

**DOI:** 10.1038/s41598-023-44195-3

**Published:** 2023-10-10

**Authors:** Ibrahim Sadiek, Gernot Friedrichs

**Affiliations:** 1 Institute of Physical Chemistry, Kiel University, Kiel, Germany; 2 Leibniz Institute for Plasma Science and Technology (INP), Greifswald, Germany; 3 Kiel Marine Science-Centre for Interdisciplinary Marine Sciences, Kiel, Germany

**Keywords:** Infrared spectroscopy, Infrared spectroscopy, Physical chemistry

## Abstract

Cross-sensitivity limits accurate quantitative detection of species concentrations in all sensor technologies, including laser-based absorption techniques. Absorption sensors capture a signal that combines contributions from all interfering species at a given detection wavelength. Careful selection of the probed spectral line, broadband detection, or upstream separation can partially mitigate cross-sensitivity, however, weak or unidentified signal interference remains a challenge for accuracy. Here, we present a proof-of-principle study to overcome cross-sensitivity by taking advantage of the distinct optical saturation characteristics of different gas mixture components. By controlling the absorption contribution of a selected species by intentional optical saturation, simultaneous and quantitative detection of two interfering species becomes possible even without the need for spectral scanning, hence offering two species–one wavelength detection (2S1W) capability. Demonstrated with direct absorption and cavity-ringdown setups, the method offers a new, previously unexploited opportunity to further enhance laser-based analyzers for complex gas mixture analysis in environmental, medical, and technical applications.

## Introduction

Absorption based quantitative detection of trace gases involves the measurement of the attenuation of a light beam transmitted through a sample as function of photon energy^1–4^. In the linear absorption regime, Beer-Lambert’s law can be directly used to determine the absolute concentration of the absorbing species, provided that the absorption cross-section is known. However, in practical applications such as the measurement of trace gases in complex gas mixtures (e.g., atmosphere, exhaust gas streams, human breath), the spectral overlap of the various mixture components results in the so-called absorption cross-sensitivity that often critically limits the performance of the analyzer. Despite the possibility to carefully select the spectral detection window, even for narrow spectral linewidth laser-based spectrometers the overlap of weak absorption features compromises the attainable species selectivity and accuracy of the detection system. Therefore, upstream separation of the sample mixture components is often necessary^5–7^, adding extra complexity and requiring regular instrumental calibration by repeated analysis of reference samples. Alternatively, broadband detection can be used together with spectral fitting routines or neural network algorithms^8^ to retrieve the mixture composition more reliably. Ever refined implementations based on incoherent and supercontinuum light sources have found widespread applications for atmospheric trace gas sensing, breath analysis, and industrial gas monitoring systems^9^. Offering a unique combination of broadband coverage and the high spectral resolution, a currently very active field of research is the development and testing of optical frequency comb spectrometers^10–12^. However, compared to proven, mostly diode laser based trace gas sensors^13,14^, field deployment of broadband detection techniques is still often hampered by limited attainable sensitivity and susceptibility to mechanical vibrations. Another possibility to deal with unwanted cross-sensitivty issues—although not fully exploited for quantitative trace gas sensing applications as yet—is to intentionally suppress the unwanted signal response of the interfering species by physical means. For example, it has been demonstrated that modulated electrical and magnetic fields can be used to restrict signal generation to neutral molecules with permanent electric (e.g., Stark modulation spectroscopy^15^) or magnetic dipole moment (e.g., Faraday rotation spectroscopy^16^). Herein, we report an alternative, more general approach that utilizes the optical saturation effect to enable quantitative detection of two species with strongly overlapping absorption lines. It is demonstrated that it is possible to decouple interfering absorptions of two species with the detection laser kept at a constant frequency (i.e., two species–one wavelength detection, 2S1W detection) by probing the overlapping transitions at variable optical saturation levels. The new technique can overcome (or simply check for) the interference of saturable weak absorptions (often caused by a minority species) or, conversely, can suppress unwanted saturable strong absorptions (often caused by a majority species) in the gas mixture. As such, the 2S1W scheme holds the potential to significantly improve the performance of laser-based trace gas sensors. Envisioned application examples are the accurate determination of isotopic signatures of trace gases (that are often hampered by tiny interfering absorption lines of other species) or the environmental detection of very low mixing ratio level trace gases (e.g., halogenated volatile compounds) without the need to remove strongly absorbing majority species (often CO$_2$, CH$_4$, or H$_2$O) from the atmospheric air samples beforehand.

Optical saturation of absorption transitions is a well-known effect in laser spectroscopy and has been mainly used as an extremely powerful tool to push the frequency precision far below the limit set by Doppler broadening. As such, the term “saturation spectroscopy”  typically alludes to experimental schemes aiming for higher spectral resolution (e.g., Lamb-dip spectroscopy^17–19^) rather than for quantitative detection of trace gas concentrations as presented in this work. On the contrary, optical saturation effects are usually considered as a disturbing factor that should be carefully avoided to ensure the validity of Beer-Lambert’s law, for example a critical issue in cavity-ringdown detection schemes^20–22^. Although somewhat different in its practical implementation, another broad field of exploiting saturation effects is in nuclear magnetic resonance, where partial saturation is intentionally induced to simplify spectral assignment. For example, partial saturation is key for nuclear overhauser effect spectroscopy^23^ and for saturation transfer difference nuclear magnetic resonance^24^.

## Results

### Partial optical saturation

 For two overlapping absorption transitions under saturation conditions, the effectively measured absorption coefficient, assuming homogeneously broadened absorption profiles, becomes a function of the input light power *P*,

1 $$\alpha (\nu , P) = \frac{\alpha ^{\textrm{gas1}}_0(\nu )}{1+P/P^{\textrm{gas1}}_{\textrm{s}}}+\frac{\alpha ^{\textrm{gas2}}_0(\nu )}{1+P/P^{\textrm{gas2}}_{\textrm{s}}}.$$

Here, $\alpha ^{\textrm{gas1}}_0(\nu )$ and $\alpha ^{\textrm{gas2}}_0(\nu )$ are the non-saturated absorption coefficients of the interfering gases (i.e., measured under linear absorption conditions), $P^{\textrm{gas1}}_{\textrm{s}}$ and $P^{\textrm{gas2}}_{\textrm{s}}$ are their corresponding saturation powers. Note that for inhomogeneous absorption profiles the denominator of Eq. (1) needs to be replaced by $\sqrt{1+P/P_{\textrm{s}}}$ and that convoluted profiles such as a Voigt function require a more detailed treatment^25,26^. The input light power *P* controls the depletion efficiency of the ground state population, while the saturation power $P_{\textrm{s}}$ is proportional to the overall relaxation rate of the excited state as well as the absorption transition probability that is related to the Einstein-*A* coefficient of spontaneous emission. As such, $P_{\textrm{s}}$ is a molecule- and collider-specific property, and it depends on the pressure as well as the mixing ratios of molecular species in the sample. Starting from low *P* values, the measured effective absorption coefficient $\alpha (\nu , P)$ for two overlapping transitions will show a ”double-bended” behavior with increasing laser power. Whereas at low laser power the absorption coefficient corresponds to the sum of $\alpha ^{\textrm{gas1}}_0$ and $\alpha ^{\textrm{gas2}}_0$, a first decrease in $\alpha$ will be observed due to the onset of saturation of the easier to saturate absorber (i.e., transition from the linear to a partial saturation regime). In case of $P^{\textrm{gas1}}_{\textrm{s}} \gg P^{\textrm{gas2}}_{\textrm{s}}$, $\alpha$ will level to a plateau corresponding to $\alpha ^{\textrm{gas1}}_0$. A further increase of *P* causes a second bend, indicating the saturation of the other saturable absorber, finally yielding complete optical saturation, $\alpha = 0$, at $P \gg P^{\textrm{gas1}}_{\textrm{s}}$. The clear observation of a double-bended absorption-power profile depends on the ratio of the saturation powers of the two overlapping transitions. For molecules with comparable transition and energy transfer probabilities, hence comparable $P_{\textrm{s}}$ values, the onset of the saturation of the two species will be hardly separable. In an ideal 2S1W experiment, $P^{\textrm{gas1}}_{\textrm{s}}$ and $P^{\textrm{gas2}}_{\textrm{s}}$ should therefore be very different to ensure a high saturation level contrast.

**Figure 1:**
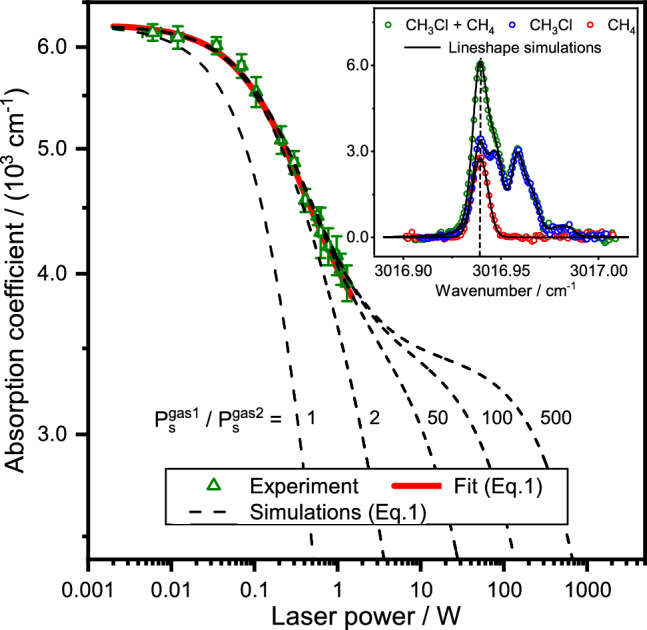
Absorption saturation. The triangles represent measured absorption coefficients for a mixture of (1.85 ± 0.05) mbar CH$_3$Cl (gas1) and (20 ± 0.5) μbar CH$_4$ (gas2) in 10 mbar Ar as buffer gas. The inset presents the measured absorption profiles of the individual gases and of the gas mixture under linear absorption conditions, i.e., at low laser power of < 4 mW. The vertical line in the inset indicates the spectral position where the data were collected during the saturation absorption experiment. The red curve represents a fit of Eq. (1) to the experimental data using $\alpha ^\mathrm {CH_4}_0$, $\alpha ^\mathrm {CH_3\textrm{Cl}}_0$, $P^\mathrm {CH_4}_{\textrm{s}}$, and $P^\mathrm {CH_3Cl}_{\textrm{s}}$ as the adjustable parameters. The dashed curves represent simulations of the double-bended saturation curves assuming different $P^{\textrm{gas1}}_{\textrm{s}}$/$P^{\textrm{gas2}}_{\textrm{s}}$=$P^\mathrm {CH_3Cl}_{\textrm{s}}$/$P^\mathrm {CH_4}_{\textrm{s}}$ ratios (with $\alpha ^\mathrm {CH_4}_0$, $\alpha ^\mathrm {CH_3\textrm{Cl}}_0$, $P^\mathrm {CH_4}_{\textrm{s}}$ fixed at their fitted values).

### Direct absorption measurements

 As proof-of-concept, a set of blended rotational-vibrational transitions of methane, CH$_4$ (gas2: easy to saturate absorber, low $P_{\textrm{s}}$), and methyl chloride, CH$_3$Cl (gas1: difficult to saturate absorber, high $P_{\textrm{s}}$), have been used as a spectroscopic model. Figure 1 presents the results of a direct absorption spectroscopy (DAS) measurements with the detection frequency set to the top of the two overlapping rovibrational transitions around 3016.94 cm$^{-1}$ (see inset of Fig. 1). A schematic of the DAS setup is shown in Supplementary Fig. S1 and representative complete spectral scans performed at different input powers are provided in Supplementary Fig. S2. As shown in Fig. 1, a first bend in the absorption-power profile already appears at a relatively low input power of about 50 mW, attributable to the onset of CH$_4$ saturation. The absorption coefficient data (triangles) were fit using Eq. (1) with $\alpha ^\mathrm {CH_4}_0$, $\alpha ^\mathrm {CH_3\textrm{Cl}}_0$, $P^\mathrm {CH_4}_{\textrm{s}}$, and $P^\mathrm {CH_3Cl}_{\textrm{s}}$ as adjustable parameters. The extracted $\alpha ^\mathrm {CH_4}_0/$ $\alpha ^\mathrm {CH_3\textrm{Cl}}_0$ absorption ratio of (0.84 ± 0.04) was found to be in very good agreement with independent linear measurements of the individual species within 2.5%. We note that the model was not able to yield a reliable value for $P^\mathrm {CH_3Cl}_{\textrm{s}}$ since the saturation of CH$_3$Cl was not fully captured by the available maximum laser power. However, the data were best reproduced by assuming a saturation power ratio of $\approx$ 50, which is consistent with the about one order of magnitude lower transition probability of CH$_3$Cl (based on Einstein-*A* coefficients tabulated in the HITRAN database^27^) and a more than one order of magnitude higher relaxation rate of CH$_3$Cl relative to that of CH$_4$^28^.

The dashed curves in Fig. 1 present simulations of double-bended absorption-power profiles using Eq. (1). Here, $\alpha ^\mathrm {CH_4}_0$, $\alpha ^\mathrm {CH_3\textrm{Cl}}_0$, and $P^\mathrm {CH_4}_{\textrm{s}}$ were kept fixed at their fitted values and $P^\mathrm {CH_3Cl}_{\textrm{s}}$ was set to $P^\mathrm {CH_3Cl}_{\textrm{s}}= n \times P^\mathrm {CH_4}_{\textrm{s}}$ with $n=1,2,50,100$, and 500. The aim of this exercise was to estimate the saturation power ratio that is required for observing a double-bended profile as a prerequisite for efficient parameters extraction. For $P^{\textrm{gas1}}_{\textrm{s}} / P^{\textrm{gas2}}_{\textrm{s}} = 1$, the absorption-power profile does not reveal a double-bended feature but rather is similar to the absorption-power profile expected for a single species. A distinct double-bended profile requires a saturation power ratio of $n \gtrsim 20$, with $n =100$ the effect is already very pronounced.

### Cavity ringdown measurements

 A cavity ringdown spectroscopy (CRDS) setup (see Fig. S1) was used as a second detection scheme to demonstrate the feasibility of the 2S1W approach. Next to the high sensitivity of CRDS for quantitative, time-resolved, and broadband trace gas detection offering a low limit of detection (LOD)^22,29,30^, placing the gas sample within a high-finesse optical cavity combines several advantages with regard to the 2S1W saturation approach: (i) During the ringdown event (i.e., the decay of light intensity within the cavity observed after switching-off the input laser), the intracavity power decays within a few tens of μs, going along with a continuous change from saturated to non-saturated conditions within a single ringdown event. Consequently, the 2S1W measurement can be performed intrinsically fast. (ii) In case of perfect coupling of the input laser power, the optical cavity can effectively multiply the input laser power by up to a factor of $2/(1-R)$, with *R* being the mirror reflectivity^31^. Hence, due to the high achievable intracavity power, 2S1W saturation experiments will become possible even with relatively low power light sources, e.g., quantum cascade diode lasers. (iii) The measurement of decay rates rather than the absolute laser intensities allows for a better constrained data fit with low uncertainties using well-developed models for the analysis of the saturated ringdown transients^26,32,33^, consequently enabling an automatized real-time data evaluation. (iv) Finally, the high sensitivity of CRDS makes it possible to work at overall low absorber densities in the buffer gas matrix, which is particularly interesting as it simplifies the energy transfer dynamics in the gas mixture. Consequently, it is more straightforward to ensure that the relaxation dynamics (and with it $P_{\textrm{s}}$) is dominated by collisions with the buffer gas (see Supplementary Note 1).

Despite these advantages, it must not be overlooked that the modeling of the ringdown event is more involved than for a simple direct absorption experiment. When two absorbing molecules with different degree of saturation are present inside an optical resonator, the decaying intracavity power reflects the saturation evolution of the absorbing molecules relative to the empty cavity decay. Indeed, the decay transient in a Saturated-CRDS (SCAR) experiment can be seen as a convolution of the decays related to the empty cavity and the absorbing gases inside the cavity. At the beginning of the ringdown event, the transition of the molecule with the lower saturation power will saturate (i.e., its absorption is “switched-off”), so that the ringdown decay will be initially determined by the empty cavity losses plus the absorption loss from non-saturated species. Later on, due to the increasing absorption contribution of the initially saturated species, the observed decay constant becomes faster and at low intracavity light power the linear absorption behavior will be restored for both species. Consequently, in the *adiabatic* limit (i.e., the overall relaxation rate is fast compared to the empty cavity decay rate, hence keeping the saturation level in a quasi-steady state during the ringdown event), the absorption of the two transitions can be decoupled by proper modeling of the observed ringdown transient. Finally, as CRDS can be considered as an absorption experiment with two counter-propagating light beams, subtle effects such as Lamb-dips may occur in the center of a Doppler-broadened absorption line profile^34^. By fitting a single exponential function to the ringdown decay transient with only methane in the cavity, we actually observed Lamb-dips that led to a reduced line center absorption signal of about 5% (see Supplementary Fig. S3).

For the analysis of the resulting ringdown transients (see Supplementary Fig. S4), we strongly rely on the saturation model put forward by Giusfredi et al.^26,32^ and also investigated by us in previous work^33^. An extended model of the intracavity power *P*(*t*) accounting for the gas absorption of two interfering species takes the general form,

2 $$\begin{aligned} P(t) = P_0 \times \textrm{exp}(-\gamma _{\textrm{empty}} t)\times \left[ f_1(t, \gamma _{\textrm{empty}}, \gamma _{\textrm{gas1}}, P_0, P^{\textrm{gas1}}_{\textrm{s}}) + f_2(t, \gamma _{\textrm{empty}}, \gamma _{\textrm{gas2}}, P_0, P^{\textrm{gas2}}_{\textrm{s}})\right] , \end{aligned}$$

with functions $f_1$ and $f_2$ describing the saturation evolution of gas1 and gas2 with $\gamma _{\textrm{gas1}} = c \alpha _0^{\textrm{gas1}}$ and $\gamma _{\textrm{gas2}}= c \alpha _0^{\textrm{gas2}}$ representing the linear gas absorption and $\gamma _{\textrm{empty}}$ the empty cavity decay constants (*c* is the light speed, see Supplementary Note 2 for further details). In this work, we exploit the practical case where gas2 has significantly lower saturation power than gas1 and where the initial light intensity is kept low enough to prevent noticeable saturation of gas1. This reduces Eq. (2) to the standard saturated-absorption ringdown equation of SCAR spectroscopy^32^,

3 $$\begin{aligned} P(t) = P_0 \times \textrm{exp}(-\gamma _1 t)\times f(t, \gamma _1, \gamma _2, P_0, P_{\textrm{s}}), \end{aligned}$$

but with $\gamma _1 = \gamma _{\textrm{empty}} + \gamma _{\textrm{gas1}}$ corresponding to an overall “non-saturated channel” and $\gamma _2 = \gamma _{\textrm{gas2}}$ to an overall “saturated channel”. Without this assumption, the measured decay transients would need to be fitted by adjusting all three beating decay constants, which turned out to be numerically unstable in some cases. The necessary temporal separation of the fitting parameters can be satisfied more easily in case of Eq. (3), where $P_0$ is set by adjusting the input laser power and $P_{\textrm{s}}$ can be tuned within certain limits by adjusting the cell pressure (i.e., the relaxation rate of the absorbing species).

**Figure 2:**
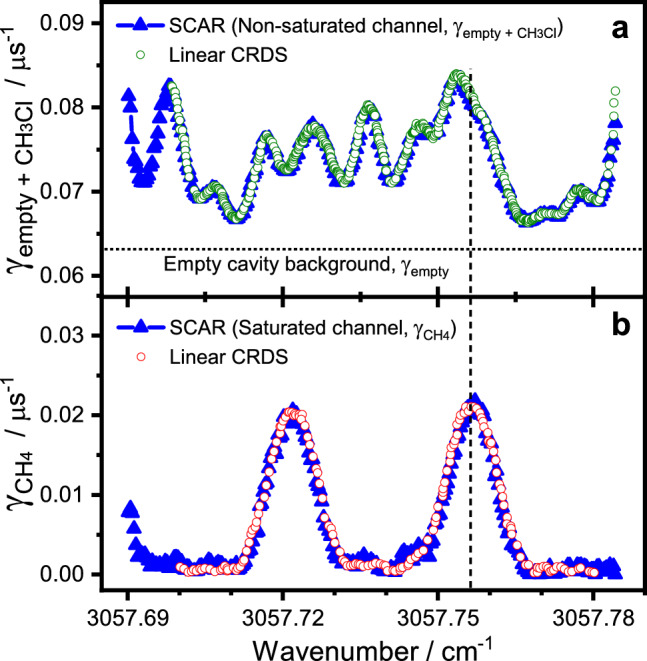
Two species–one wavelength (2S1W) demonstration. The triangles represent the decoupled high-resolution spectra of CH$_3$Cl (a, non-saturated channel) and CH$_4$ (b, saturated channel) from fitting measured decay transients with Eq. (3) using $\gamma _1$, $\gamma _2$, and $P_{\textrm{s}}$ as adjustable parameters. Gas mixture: 30 μbar CH$_3$Cl and 8 nbar CH$_4$ in 2.50 mbar Ar at room temperature. Measurement parameters: 1.6 W intracavity power, 50 times averaged transients, 5 Hz repetition rate. The open circles depict reference absorption spectra for CH$_3$Cl in Ar (green) and CH$_4$ in Ar (red), recorded under linear absorption conditions with the same CRDS setup. The dashed vertical line denotes the absorption transition used for the linearity check shown in Fig. 3.

Figure 2 presents the outcome of a SCAR scan across overlapping transitions of CH$_3$Cl and CH$_4$ at wavelengths around 3057.73 cm$^{-1}$. The scan has been performed under saturation conditions with an intracavity light power of about 1.6 W (for details of the evaluation of the intracavity power see Supplementary Note 3). The laser was slowly scanned over the absorption feature and the ringdown signals were analyzed on-the-fly by fitting Eq. (3) to the decay transients (for details of the actual fit model and procedure see Supplementary Note 2). The extracted $\gamma _1 = \gamma _{\textrm{empty}} + \gamma _\mathrm {CH_3Cl}$ and $\gamma _2 = \gamma _\mathrm {CH_4}$ decay constants are depicted in Fig. 2a (non-saturated channel) and Fig. 2b (saturated-channel) as blue triangles, respectively. The individual reference spectra of CH$_4$ and CH$_3$Cl, measured with the same CRDS setup under linear absorption conditions at very low intracavity light power, are included in the same panels as open circles. Both the two-species SCAR and the one-species CRDS spectra match very well, clearly showing the separability of the two decay constants under saturation conditions. This experiment highlights the key advantage of the 2S1W approach, i.e., the possibility to extract absorption of a target species even in the presence of strongly interfering absorption from a second species. Whereas the absorption of the saturable absorber (here CH$_4$) can be determined without knowledge of the empty cavity decay constant, in order to extract the absorption of the non-saturated species (here CH$_3$Cl), the empty cavity decay constant needs to be determined independently (as is the case for a linear CRDS experiment as well). Closer inspection of Fig. 2 reveals that the noise level in the extracted $\gamma _\mathrm {CH_4}$ absorption trace is somewhat higher than for $\gamma _\mathrm {empty+CH_3Cl}$. Actually, one might have expected the opposite as the empty cavity background fluctuations often represent a major source of noise in standard CRDS experiments and hence should show up in the non-saturated channel. However, in case of the 2S1W approach, the extra noise in the saturated channel is simply due to the fact that $\gamma _\mathrm {CH_4}$ is extracted from the later part of the ringdown event that corresponds to low intracavity light levels and with it an intrinsically higher noise level (see Supplementary Fig. 4b). It is important to note that no Lamb-dips are observed in the spectrum of the saturated CH$_4$ channel. This is the expected as the reported $\gamma _\mathrm {CH_4}$ values correspond to non-saturated decay constants. Instead, within the framework of our simplified model, indications for weak Lamb-dip features are seen in the saturation parameter $P_{\textrm{s}}$ at the center of CH$_4$ absorption lines (see Supplemental Fig. S5). Further analysis of this effect would have been beyond the scope of this initial proof-of-principle study, but future research should certainly focus on more refined fitting models, where more sophisticated line-shape models may help to further improve the decoupling of the various parameters.

A more detailed analysis of the performance of the current 2S1W CRD implementation in terms of Allan standard deviation of the extracted $\gamma _\mathrm {CH_4}$ and $\gamma _\mathrm {empty+CH_3Cl}$ values is shown in Supplementary Fig. S6. This analysis revealed that $\gamma _\mathrm {empty+CH_3Cl}$ exhibits about a factor of 3 lower noise compared to $\gamma _\mathrm {CH_4}$. According to the Allan plot, the optimum sampling time to achieve the lowest LOD is about 220 s (analysis of about 1100 decay transients at a sample repetition frequency of about 5 Hz, yielding a standard deviation of $0.6 \times 10^{-4}$ μs$^{-1}$ and 2.1 × 10$^{-4}$ μs$^{-1}$ for $\gamma _\mathrm {empty+CH_3Cl}$ and $\gamma _\mathrm {CH_4}$, respectively. The single-shot standard deviations of $2.3 \times 10^{-3}$ μs$^{-1}$ and 7.8 × 10$^{-3}$ μs$^{-1}$ correspond to absorption coefficients of 7.7 $\times$ 10$^{-8}$ cm$^{-1}$ and 2.6 $\times$ 10$^{-7}$ cm$^{-1}$, hence the achieved LOD compares very well with previously reported values of 0.2–1.5 $\times$ 10$^{-7}$ cm$^{-1}$ for mid-IR quantum cascade laser based CRDS implementations for single species detection in the linear absorption regime^35^. The observed excellent decoupling of the two parameters is in agreement with earlier results of Galli et al.^36^ who took advantage of the capability of SCAR to simultaneously extract $\gamma _1=\gamma _{\textrm{empty}}$ and $\gamma _2=\gamma _\mathrm {^{14}CO_2}$ from saturated ringdown decays. They used SCAR for single-species detection of $^{14}$CO$_2$ and demonstrated outstanding sensitivity for radiocarbon dating applications.

**Figure 3:**
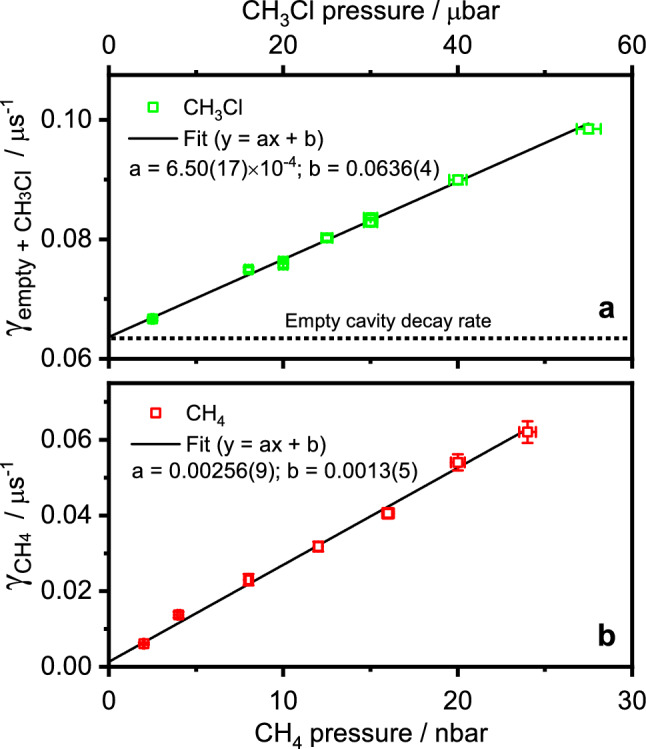
2S1W linearity. Extracted decay constants as a function of the partial pressure of CH$_3$Cl (panel a, with CH$_4$ kept constant at 8 nbar) and of CH$_4$ (panel b, with CH$_3$Cl kept constant at 30 μbar). The measurements were performed with the laser frequency set to the strongly overlapping absorption transition at 3057.756 cm$^{-1}$ (see Fig.2), Measurement parameters: about 1.7 W intracavity light power, 2.50 mbar total cell pressure. The horizontal error bars represent an estimated 2% uncertainty of the gas mixture composition, resulting from the pressure readout. The vertical error bars correspond to the standard deviation of 10–20 consecutive measurements, where each individual value was the average of 10 transients.

### Linearity

Figure 3 depicts the linearity of the extracted absorption decay constants as a function of the partial pressures of CH$_4$ and CH$_3$Cl in the gas mixtures. Two series of experiments are presented, both performed at a total cell pressure of $2.50 \pm 0.05$ mbar and an intracavity power of about 1.7 W. For experiments in panel (a), CH$_4$ was kept constant at 8 nbar and CH$_3$Cl was varied from 5 rto 55 μbar. For experiments in panel (b), CH$_3$Cl partial pressure was kept constant at 30 μbar and the CH$_4$ pressure was varied from 2 to 24 nbar. The vertical error bars represent the standard deviation of the 10–20 repeated measurements, each an average of 10 saturated CRD transients. Overall, the extracted decay constants reveal a very good linearity both with respect to CH$_3$Cl and CH$_4$ partial pressures, again highlighting the reliable decoupling of the two parameters $\gamma _1$ and $\gamma _2$.

## Discussion

We have demonstrated a new approach for quantitative detection of two molecular species with overlapping absorption transitions (two-species–﻿one wavelength detection, 2S1W) by utilizing the different optical saturation behavior of the interfering absorbers. 2S1W is a type 2D absorption spectroscopy, with the frequency axis as the first dimension and the detection light power (i.e., the sample saturation) as the second dimension. It enhances the capability of narrow bandwidth laser-based detection schemes for multi-species sensing and holds the potential to relax cross-sensitivity issues that often limit the accuracy of trace gas sensors in practical applications. In principle, provided that light sources and/or optical setups are available that allow for sufficient light intensity tuning, the optical saturation based approach can be implemented in many variants of laser absorption spectroscopy. As it is demonstrated in this proof-of-concept study, the CRDS scheme is superior to the DAS scheme in terms of fast and reliable parameter extraction, but careful choice of the experimental conditions are necessary to ensure the applicability of the SCAR fitting model.

**Figure 4:**
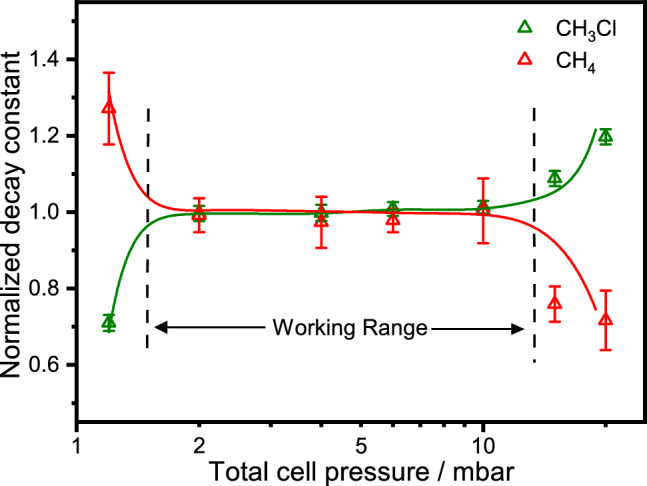
2S1W working limits. Extracted $\gamma _\mathrm {CH_4}$ (red) and $\gamma _\mathrm {empty+CH_3Cl}$ (green) as a function of the total cell pressure. The decay constants are normalized with respect to those obtained from independent measurements under linear absorption conditions. The solid curves reflect the overall trend. Measurements were performed with the laser frequency set to the strongly overlapping absorption transition at 3057.756 cm$^{-1}$ (see Fig.2). Gas mixture: 30 μbar CH$_3$Cl, 8 nbar CH$_4$, and Ar as the buffer gas.

The working limits of the saturation model of Eq. (3) for 2S1W detection were tested by extending the analysis of saturated CRD transients over a wider dynamic range of cell pressures, hence variable saturation powers $P_{\textrm{s}}$. Figure 4 shows the extracted decay constants for the overlapping CH$_4$/CH$_3$Cl transitions at intracavity light power of about $1.6$ W as a function of the total cell pressure. The extracted $\gamma _\mathrm {CH_4}$ and $\gamma _\mathrm {CH_3Cl}$ were normalized with respect to their non-saturated values obtained from independent CRDS measurements in the linear absorption regime at each cell pressure. Hence, the unity values in Fig. 4 indicate a reliable parameter extraction for pressures ranging from 2.0 mbar to 10 mbar. However, starting at a pressure of 15 mbar, the saturation of CH$_4$ decreases due to increasingly fast relaxation and the model starts to underestimate the corresponding decay constant of the saturated channel. Correspondingly, a pronounced overestimation of the non-saturated $\gamma _\mathrm {empty+CH_3Cl}$ value takes place. Just vice versa, at a pressure of 1.2 mbar, $\gamma _\mathrm {empty+CH_3Cl}$ is already underestimated by as much as 30% because saturation of CH$_3$Cl takes effect.

The deviations at high and low pressures define the working range of the simple 2W1S fitting approach based on Eq. (3). The actual working range will depend on the studied molecular system, but within certain limits it is possible to tune the experimental conditions to be compatible with the 2S1W approach. For example, the working range will shift towards lower or higher pressures depending on the saturation powers of the overlapping transitions as well as the intracavity power. Increasing the intracavity power will shift the working range towards higher pressures, while a higher saturation power will shift it towards lower pressures. Note that also the choice of the buffer gas may allow species-selective tuning of the saturation powers in favorable cases because collisional energy transfer dynamics is a species-specific process.

In practice, next to a proper selection of absorption transitions that offer high saturation contrast, suitable experimental conditions for a 2S1W experiment can be identified by a preliminary analysis of less congested parts of the interfering absorption spectra. As an example, Supplementary Fig. S7 illustrates a scan over three absorption lines, covering one strongly overlapping CH$_4$/CH$_3$Cl transition and two isolated “non-saturated” absorption lines of CH$_3$Cl. Here, it turned out that a small portion of the CH$_3$Cl absorption signal showed up in the saturated channel, which is due to the onset of weak saturation of CH$_3$Cl. Fully consistent with this finding, the amplitude of the CH$_4$ signal in the “saturated” channel of the overlapping line was somewhat overestimated. In such a case, slightly increasing the pressure would help to recover the full decoupling of the two-species absorption spectra.

## Outlook

Future work, next to the implementation of improved lineshape models and fitting strategies, will focus on the demonstration of a 2S1W experiment for analyzing environmental gas samples. Moreover, the role of energy transfer and buffer gas pressure to modulate the saturation powers of the interfering species shall be investigated in more detail. Supplementary Note 1 and Fig. S8 (both highlighting details of the energy-transfer dynamics in the CH$_3$Cl/CH$_4$ molecular system) as well as Supplementary Table S1 (listing atmospheric molecules grouped in terms of their self-collision relaxation rates) may serve as a starting point for steps into these directions.

A key aspect will be to further develop robust fitting strategies to extract the absorptions of two partially saturated species (i.e., dropping the applied approximation that one species is not saturated at all), for example by direct application of Eq. (2) for fitting the SCAR transients. Another interesting extension of the method would be to measure and analyze saturated spectra of a gas mixture with two, three, or more species at different light intensities and over a wider spectral range. Using the detection light intensity as a second axis, such measurements could lay the foundations of a new type of 2D spectroscopy, namely 2D-LASSS (Laser Absorption Selective Saturation Spectroscopy), where a global fit of overlapping spectra (1st axis) with different extent of saturation (2nd axis) may allow for an improved and quantitative deconvolution of the individual species contributions.

## Methods

### Materials

 Gas mixtures of methane, CH$_4$ (99.5%), and methyl chloride, CH$_3$Cl (99.8%), were used as a model molecular system. Argon, Ar (99.999%), served as the buffer gas. Several overlapping rovibrational transitions of the $\nu _3$ fundamental band of CH$_4$ and the $\nu _4$, 2$\nu _6$, and $\nu _1$ bands of CH$_3$Cl were investigated in the wavenumber range 3016–3058 cm$^{-1}$. All measurements were carried out at room temperature.

### Direct absorption spectroscopy (DAS)

 A schematic of the experimental layout of the direct absorption spectrometer is shown in Supplementary Fig. S1. A continuous-wave, single resonant optical parametric oscillator (cw-SR-OPO, Lockheed-Martin, Aculight Argos 2400-SF), pumped by 10 W of a fiber-amplified (IPG Photonics) Yb-doped DFB fiber laser (NKT Photonics) operated at 1064 nm, has been used as the light source. The cw-SR-OPO light source was capable of producing >1 W of tunable idler output between 3.2 and 3.9 μm, with a beam diameter of about 3.0 mm. A wavemeter (Bristol Instruments, 621A-IR) with an accuracy of $\pm 0.0006$ cm$^{-1}$ at 3.3 μm was used for wavelength measurements. The incident laser beam passed the measurement cell (15 cm long, fused silica windows) without any focusing, while the transmitted light was focused by a 10 cm focusing lens placed in front of the preamplified liquid nitrogen cooled InSb photodiode detector (SVS-Vistek, KA-05-CI, 5 MHz bandwidth). A second thermo-electrically cooled HgCdTe preamplifier/photodiode combination (Vigo System, PVI-2TE-5-0) was used as a reference detector for continuous monitoring of the input laser intensity.

### Cavity ringdown spectroscopy (CRDS)

 A schematic of the experimental layout of the cavity-ringdown spectrometer is shown in Supplementary Fig. S1, a more detailed description can be found in our previous paper^33^. A Fabry-Perot resonator with a mirror separation of $51$ cm, equipped with ringdown mirrors with a radius of curvature of 100 cm, has been used. Two mode matching lenses were placed before the entrance mirror to improve the geometrical overlap of the laser beam with the TEM$_{00}$ mode of the resonator. The same cw-SR-OPO and wavemeter as described for the DAS experiment have been used as the light source. An acoustic optical modulator (AOM, Gooch & Housego, 20 ns rise time) served as a fast optical switch to cut the excitation light after the intracavity light power had reached a pre-set trigger level. Frequency matching of the optical cavity was achieved by modulating the cavity length using three piezo electric transducers acting on one of the cavity mirrors, resulting in a scanning speed of the cavity resonance frequency of 15 GHz/s. A liquid nitrogen cooled InSb photodiode/preamplifier combination (SVS-Vistek, KA-05-CI, 5 MHz bandwidth) was used for detecting the decaying transmitted light behind the optical cavity. The actual measured ringdown time of the empty cavity was about 16 μs, corresponding to an effective absorption pathlength of 4.8 km. Ringdown transient were captured at a repetition frequency of about 5 Hz.

### Data acquisition

 The collection of the ringdown transients as well as the continuous monitoring of the laser wavelength were accomplished with a high-resolution flexible digitizer (National Instrument, NI 5922, 10 MS/s, 18 bit) and home-written LabView software. Each individual ringdown signal was digitized for a time interval extending about 10 ringdown decay times. The nonlinear programming solver *fmincon* implemented in MATLAB software has been used as a fitting routine to extract the parameters $\gamma _1$ and $\gamma _2$ along with other parameters (see also Supplementary Note 2 for further details). $\gamma _1$ and $\gamma _2$ encode the gas absorption decay constants of the overlapping transitions as well as the empty cavity decay constant. Typically, five consecutive ringdown transients were averaged prior to numerical fitting. The same digitizer and a home-written LabView code were also used for the data acquisition with the DAS setup.

### Supplementary Information

Supplementary Information.

## Data Availability

The data that support the findings of this study are available from the corresponding author upon reasonable request.
