# Supplementary material for: Two species–one wavelength detection based on selective optical saturation spectroscopy
Source: Sci Rep. 2023 Oct 10;13:17098. doi: 10.1038/s41598-023-44195-3 (PMC10564745; doi:10.1038/s41598-023-44195-3)
Supplement: Supplementary file 1 — Supplementary Information. [file 41598_2023_44195_MOESM1_ESM.pdf]

Supplementary Material

Two species - one wavelength detection based on selective optical saturation spectroscopy

Ibrahim Sadiek,[1,2] Gernot Friedrichs[1,3,*]

[1]Institute of Physical Chemistry, Kiel University, Kiel, Germany
[2]Leibniz Institute for Plasma Science and Technology (INP), Greifswald, Germany
[3]Kiel Marine Science - Centre for Interdisciplinary Marine Sciences, Kiel, Germany

[*]To whom correspondence should be addressed; E-mail: friedrichs@phc.uni-kiel.de

- Supplementary Notes
  - Note S1: Energy Transfer Dynamics
  - Note S2: Saturation Model
  - Note S3: Coupling Efficiency and Intracavity Power
- Supplementary Figures
  - Figure S1: DAS & CRDS setup
  - Figure S2: DAS spectra
  - Figure S3: Lamb-dip feature
  - Figure S4: SCAR decay transients
  - Figure S5: SCAR decay transients
  - Figure S6: SCAR fit example
  - Figure S7: 2S1W cross-sensitivity
  - Figure S8: CH_3Cl/CH_4 energy transfer model
- Supplementary Tables
  - Table S1: V-T relaxation times

Supplementary Notes

Note S1: Energy-Transfer Dynamics

The energy-transfer dynamics of the molecular system directly influence the optical saturation power and as such is an important aspect for designing the optimal measurement conditions for a 2S1W experiment.

Resonant absorption of laser photons in the infrared spectral region typically excites molecules from the ground to their excited vibrational states. In addition, molecular collisions populate and depopulate rotational sublevels within the ground and the excited state manifolds of the connected states. Following vibrational excitation, the deactivation is ultimately controlled by the conversion of the vibrational energy content to translational energy of the bath gas (i.e., V-T transfer) [1]. This energy dissipation is usually effective from the lowest vibrational modes that are populated after a series of intramolecular vibrational-vibrational (V-V) energy transfer events between the different vibrational manifolds. Additionally, collisional deactivation can occur by a quenching mechanism initiated by collision with another relaxing molecule via near-resonant intermolecular V-V transfer. In cases where the vibrational energy transfer from molecule A to molecule B is accompanied by a very efficient V-T transfer from molecule B to the bath gas, the time scale of the relaxation of molecule A may be determined by the V-T transfer from molecule B.

Supplementary Fig. S8 presents a schematics of a vibrational energy level diagram for a mixture of the two polyatomic relaxing gases A (here, CH_4) and B (here, CH_3Cl). In the 2W1S scenario with overlapping rovibrational absorption transitions, the vibrational transitions of the two gases are nearly resonant. Therefore, fast near-resonant V-V transfer between vibrationally excited A* and B (or vice versa) is very likely to occur. As a consequence, fast equilibration of A* and B* may take effect. Now let us assume for a mixture of A and B that (i) the equilibrium population of A* and B* scales with their respective mole fractions x_A and x_B and (ii) the lowest vibrational mode of B is lower than the lowest vibrational mode of A such that the V-T transfer of B can be assumed to be faster than that of A. In this scenario, the effective relaxation time $\beta^\mathrm{eff}_\mathrm{A^*}$ is governed by $1/\beta^\mathrm{eff}_\mathrm{A^*} = (1 - x_\mathrm{B})/\beta_\mathrm{A^*} + x_\mathrm{B}/\beta_\mathrm{B^*}$, where $\beta_\mathrm{A^*}$ and $\beta_\mathrm{B^*}$ are the overall relaxation times found in pure gas samples of A and B, respectively. At sufficiently high x and with $\beta_\mathrm{A^*} > \beta_\mathrm{B*}$, the relaxation of A* becomes completely dominated by $\beta_\mathrm{B^*}$. Under such conditions, both A* and B* will show almost the same relaxation times and hence also their saturation powers may become very similar. Consequently, the necessary decoupling of the saturated channel, γ_1, and non-saturated channel, γ_2 may not be possible.

However, with decreasing mixing ratio of A and B in the sample gas mixture (e.g., by diluting the sample with a buffer gas or by directly analyzing trace gases in atmospheric air samples), species A and B will start to relax independently from each other, simply due to the quadratic decreasing probability of a resonant V-V energy transfer collision. Therefore, for practical 2S1W implementations, the combination of a sensitive CRDS approach with diluted gas samples is advantageous in two respects. First, the mole fraction of the species can be kept low enough to ensure independent relaxation pathways of the interfering species and, secondly, buffer gas dominated relaxation pathways ensure that the extracted P_s values do not depend on the mole fractions of the absorbing gases. The latter is not a necessary condition for a 2S1W

analysis (because P_s can be treated a free-floating parameter), but contributes to a more stable fit result.

From this brief description it is clear that a successful 2S1W detection largely depends on different vibrational relaxation times of the two interfering species. To range the applicability of the 2S1W approach, Table S1 lists atmospherically important trace gases classified with respect to their relaxation times β, which are closely related to the saturation power of the different species. Note that merely vibration-translation energy transfer relaxation times for self-collisions are reported in Table S1 and that the corresponding transition probabilities of the probed optical transitions also determine the saturation powers. Therefore, a simple comparison based on the listed relaxation times only serves as a first rough guideline to identify potentially promising trace gas pairs. For example, the measurement of ammonia in the presence of CO_2 should be straightforward since the the relaxations times are different by more than four orders of magnitude. Similarly, many halogenated methanes (CH_3I, CH_2Cl_2, $CHBr_2Cl$, etc.) can be measured in the presence of CH_4, CO_2, or N_2O. Even water absorption can probably be decoupled from strong absorbing greenhouse gases such as CO_2, COS, CS_2, N_2O, and CH_3F.

Note S2: Saturation Model

The "double-bended" absorption-power profile of the direct absorption measurements under absorption saturation conditions (Fig. 1, main text) were directly fitted using Eq. 1 (main text) with $\alpha_0^{\mathrm{CH_3Cl}}$, $\alpha_0^{\mathrm{CH_4}}$, $P_\mathrm{s}^{\mathrm{CH_4}}$, and $P_\mathrm{s}^{\mathrm{CH_3Cl}}$ as fit parameters.

For the cavity ringdown measurements under absorption saturation conditions, the observed ringdown decay is determined by the convolution of the time-independent empty cavity decay and the time-dependent evolution of the gas absorption decay for the two overlapping absorption transitions. The resulting ringdown can be represented as follows:

$$\begin{aligned} P(t) = P_0 \times \exp(-\gamma_{\mathrm{empty}} t) \times \\ \left[f_1(t, \gamma_{\mathrm{empty}}, \gamma_{\mathrm{gas1}}, P_\mathrm{s}^{\mathrm{gas1}}, P_0) + f_2(t, \gamma_{\mathrm{empty}}, \gamma_{\mathrm{gas2}}, P_\mathrm{s}^{\mathrm{gas2}}, P_0) \right]. \end{aligned} \tag{S1}$$

$P(t)$ is proportional to the measured detector signal $S(t)$ with the calibration parameter $C = S/P$ (see also Note S3), hence

$$\begin{aligned} S(t) = S_0 \times \exp(-\gamma_{\mathrm{empty}} t) \times \\ \left[f_1(t, \gamma_{\mathrm{empty}}, \gamma_{\mathrm{gas1}}, Z_{\mathrm{gas1}}^{\mathrm{1V}}) + f_2(t, \gamma_{\mathrm{empty}}, \gamma_{\mathrm{gas2}}, Z_{\mathrm{gas2}}^{\mathrm{1V}}) \right] + S_{\mathrm{BG}}. \end{aligned} \tag{S2}$$

Here, S_{BG} has been added as a detector offset. As indicated in Refs. [2, 3], the saturation power P_s is related to the adjustable parameter $Z^{\mathrm{1V}} = 1/(CP_s)$ that corresponds to the value of $P(t)/P_s$ for a signal voltage amplitude of 1V.

Eq. S2 can be understood by considering the absorption-induced power attenuation of a Gaussian beam along the propagation direction z,

$$\frac{\mathrm{d}P}{\mathrm{d}z}(t) = -2\pi \int_0^\infty \alpha(\rho, t) I(\rho, t) \rho \mathrm{d}\rho, \tag{S3}$$

where $\rho = \sqrt{(x^2 + y^2)}$ is the radial distance from the z asis and $\alpha(\rho, t)$ the effective absorption coefficient for the two absorbing species. The latter is given by Eq. 1 (main text) for homogeneously (pressure) broadened absorption line profiles or its variant,

$$\alpha(\nu, P) = \frac{\alpha_0^{\mathrm{gas1}}(\nu)}{\sqrt{1 + P/P_{\mathrm{s}}^{\mathrm{gas1}}}} + \frac{\alpha_0^{\mathrm{gas2}}(\nu)}{\sqrt{1 + P/P_{\mathrm{s}}^{\mathrm{gas2}}}}, \tag{S4}$$

for inhomogeneously (Doppler) broadened absorption line profiles. For a more detailed treatment of SCAR theory that also includes the case of convoluted Voigt line profiles, we refer to the comprehensive work of Giusfredi et al. [2] and Mazzotti et al. [4]. For the sake of completeness, however, note that the saturation power P_{s} is proportional to $g^{-1}(\nu)$ for an homogeneously broadened line whereas $P_{\mathrm{s}} \propto g^{-1}(\nu_0)$ holds for an inhomogeneously broadened absorption line with lineshape function $g(\nu)$ peaking at ν_0. Using $I(\rho, t) = I_0(t)\mathrm{e}^{-2(\rho^2/\omega^2)}$ for the time-dependent intensity of a TEM_{00} mode of a laser beam and assuming that the beam waist ω can be treated as a constant along the cavity axis (this is a valid assumption since about 85% of the cavity length was within the Rayleigh range), substitution of Eq. S4 into Eq. S3 and integration yields the intracavity power attenuation due to absorption. Including the quasi-continuous mirror loss decay term, the overall power attenuation within the ringdown cavity can be expressed as:

$$\frac{\mathrm{d}P(t)}{\mathrm{d}t} = -\left(\gamma_{\mathrm{empty}} + \frac{2\gamma_{\mathrm{gas1}}}{1 + \sqrt{1 + P/P_{\mathrm{s}}^{\mathrm{gas1}}}} + \frac{2\gamma_{\mathrm{gas2}}}{1 + \sqrt{1 + P/P_{\mathrm{s}}^{\mathrm{gas2}}}}\right) P(t). \tag{S5}$$

Eq. S5 represents the general form of the SCAR model, which was first introduced by Giusfredi et al. [2, 5] for one species detection. Here, solely an additional term shows up for the second absorbing species.

As already outlined in the main text, the major challenge in using Eq. S5 for extracting the individual absorptions ($\gamma_{\mathrm{gas}\,i} = c\alpha_0^{\mathrm{gas}\,i}$) is that they are not necessarily temporally resolved from γ_{empty} (i.e., γ_{gas1}, γ_{gas2}, and γ_{empty} are more or less coupled). A more straightforward and numerically stable implementation of Eq. S5 for two species detection can be achieved by reducing the three parameters to only two, namely $\gamma_1 = \gamma_{\mathrm{empty}} + \gamma_{\mathrm{gas1}}$, and $\gamma_2 = \gamma_{\mathrm{gas2}}$.

$$\frac{\mathrm{d}P(t)}{\mathrm{d}t} = -\left(\gamma_1 + \frac{2\gamma_2}{1 + \sqrt{1 + P/P_{\mathrm{s}}^{\mathrm{gas2}}}}\right) P(t). \tag{S6}$$

This is possible for $P^{\mathrm{gas1}} > P$, which can achieved by controlling both intracavity power and gas pressure. The experimental parameters can be tuned such that gas1 is non-saturated over the whole ringdown event and gas2 is saturated at the beginnig of the ringdown event but becomes non-saturated at not too low intracavity power. Note that γ_{empty} needs to be determined independently, preferentially from a ringdown event without the presence of gas1 and gas2 or by tuning the detection laser off-resonance.

Consequently, Eq. S2 reduces to

$$S(t) = S_0 \times \exp(-\gamma_1 t) \times f(t, \gamma_1, \gamma_2, Z^{1\mathrm{V}}) + S_{\mathrm{BG}}. \tag{S7}$$

Here, $S(0) = S_0 + S_{\mathrm{BG}}$ and $f(0) = 1$. As can be directly deduced from Eq. S7 for $S_{\mathrm{BG}} = 0$, the so-called ratio function f describes the evolution of the intracavity power relative to the decay curve observed with the decay constant γ_1. This can be also seen in Fig. S4 that shows

typical experimental ringdown decay curves measured in this work.

The first derivative of the ratio function is given by

$$\frac{\mathrm{d}f(t)}{\mathrm{d}t} = -\frac{2\gamma_2}{1 + \sqrt{1 + S_0 Z^{1\mathrm{V}} \exp(-\gamma_1 t) \times f(t)}} \tag{S8}$$

in case of an inhomogeneously broadened absorption profile and by

$$\frac{\mathrm{d}f(t)}{\mathrm{d}t} = -\gamma_2 \frac{\ln\left[1 + S_0 Z^{1\mathrm{V}} \exp(-\gamma_1 t) \times f(t)\right]}{S_0 Z^{1\mathrm{V}} \exp(-\gamma_1 t)} \tag{S9}$$

for the homogeneously broadened case. Both Eq. S7 and S8 (resp. S9) are included in the fitting routine and the integration of Eqs. S8 (resp. S9) is performed numerically on-the-fly using the Runge-Kutta-Fehlberg method, RK45.

Note S3: Coupling Efficiency and Intracavity Power

Saturation experiments in direct absorption setups rely on the availability of detection lasers with high power. However, even for comparatively low power lasers sources (such as quantum cascade laser diodes), 2S1W measurements become possible by taking advantage of the intensity enhancement by optical cavities - provided that the laser beam is coupled into the high-finesse cavity with sufficiently high efficiency.

The ultimate achievable intracavity light power can be theoretically estimated based on [6]:

- the ratio of the laser to cavity mode linewidth $\kappa = \Delta\nu_{\mathrm{laser}}/\Delta\nu_{\mathrm{cavity}}$ (often $\Delta\nu_{\mathrm{cavity}} < \Delta\nu_{\mathrm{laser}}$ such that only part of the laser light couples into the cavity),
- the scanning speed of the cavity resonance (or the laser frequency) relative to the cavity resonance widths , and
- the geometrical coupling efficiency ϵ_{mode} (which accounts for the need of a good spatial overlap of the laser beam with that of the TEM_{00} mode of the cavity).

For a unity coupling efficiency and for typical ringdown mirrors with a reflectivity of $R = 0.999893$ as used in this work, an intensity enhancement factor of up to $2/(1-R) = 1.9 \times 10^4$ should arise. However, as outlined in the works of Hahn et al. [7] and Morville et al. [8], practical enhancement factors are usually much lower. In our experimental setup, $\Delta\nu_{\mathrm{laser}} \approx$ 60 kHz on a 500 μs timescale and 1MHz on a timescale of 80 ms. $\Delta\nu_{\mathrm{cavity}}$ is about 10 kHz, as estimated from the measured empty cavity decay time of 15.85 μs for the 0.51 m long Fabry-Perot cavity. With values of $\kappa = 6$ (60 kHz laser linewidth) or $\kappa = 100$ (1 MHz laser linewidth), an approximate scan speed of 15 GHz/s, and an estimated mode matching factor of $\epsilon_{\mathrm{mode}} \approx 0.5$, the resulting coupling efficiency is about 2% (60 kHz laser linewidth) and 0.5% (1 MHz laser linewidth), respectively (see Fig. 7 in Ref. [8]). This would still correspond to a light enhancement factor of 380 or 95, respectively.

The experimental intracavity power and with it the enhancement factor can be estimated through a conversion factor $C = S/P$ that relates the measured detector signal S to the intracavity power P,

$$C = R_{\mathrm{D}} \times \frac{T(1-L)}{2} \approx R_{\mathrm{D}} \times \frac{(1-R)(1-L)}{2}. \tag{S10}$$

Here, $T = 1 - R = 1.07 \times 10^{-4}$ is the mirror transmission, with the mirror reflectivity R determined from the ringdown time τ_0 = 15.85 μs and $R_{\mathrm{D}} = 1.29 \times 10^5$ V/W is the detector responsivity. L is an additional loss parameter to account for the extra losses experienced by the detected light (e.g., by CRD mirror substrate, absorption, surface reflections, etc.) before reaching the detector. Here, we set $L = 0.5$ to account for the reflection losses by a focusing lens in front of the detector and undisclosed absorption/scattering by mirror coatings, which could be even larger than the mirror transmission in the mid-IR region [9]. Using Eq. S10, a typically measured 4.0 V detector signal from 50 mW of incident power on the cavity first mirror would correspond to an intracavity power of 1.3 W, hence an enhancement factor of 26. While being high enough for the saturation experiments, this is nevertheless significantly lower than the theoretical estimate outline above. Next to a possible underestimation of the loss factor L or overestimation of the geometrical factor (e.g., as a result of imperfect TEM_{00} excitation of the cavity), we also attribute this remaining discrepancy to noise of the piezo amplifier used to drive the scan piezo of the OPO seed laser. This noise directly translate into frequency noise of OPO light output such that it is not fully clear, which effective laser linewidth to use for the theoretical estimate.

Supplementary Figures

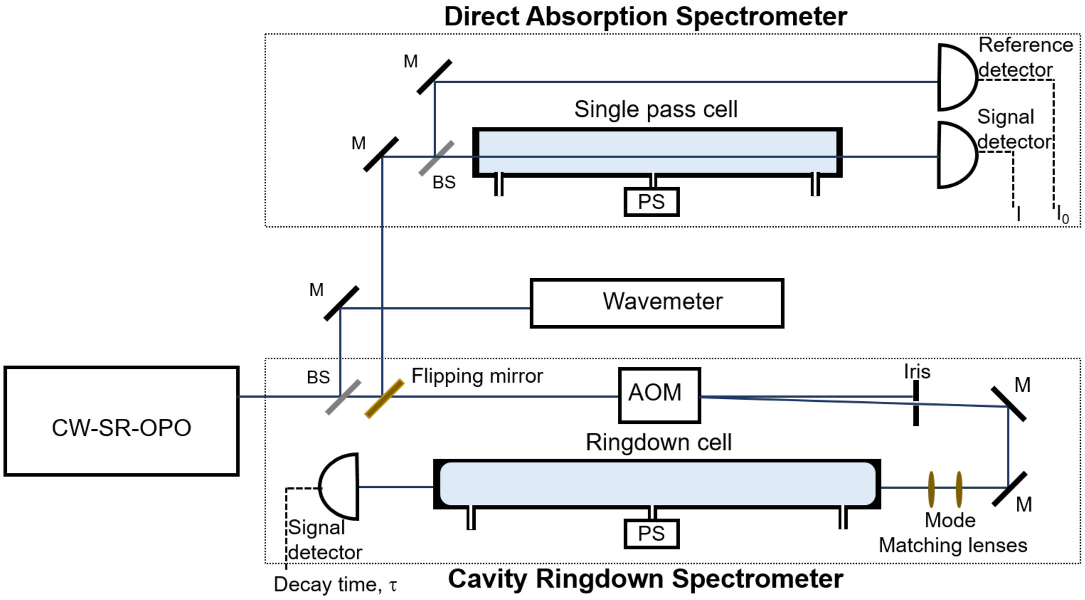

Figure S1: DAS and CRDS setup. Schematic diagram of the direct laser absorption spectroscopy (DAS) setup (upper) and the cavity ringdown spectroscopy (CRDS) setup (lower). The same continuous wave-single resonant- optical parametric oscillator (CW-SR-OPO) was used as the light source. AOM: acousto-optic modulator, BS: beam splitter, M: mirror, PS: pressure sensor.

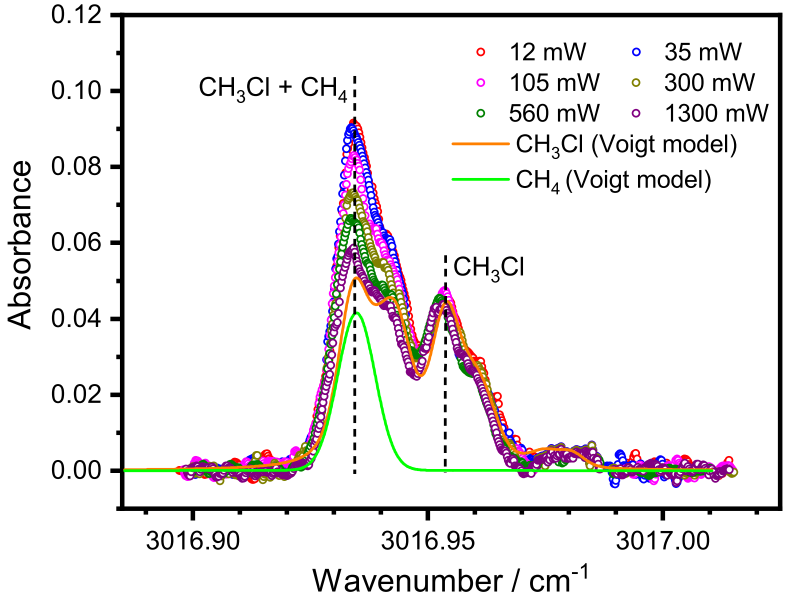

Figure S2: DAS spectra. Absorbances for a mixture of (20 ± 0.5) μbar CH_4 and (1.85 ± 0.05) mbar CH_3Cl in 10 mbar Ar, measured at variable input laser power. The 2 times averaged spectra (symbols) were obtained by a continuous slow scan of the detection laser wavelength. The simulated individual spectra of CH_3Cl and CH_4 (solid curves) are based on a Voigt lineshape function and HITRAN data [10]. As the laser power increases, the CH_4 absorption saturates and the measured spectrum becomes dominated by CH_3Cl absorption.

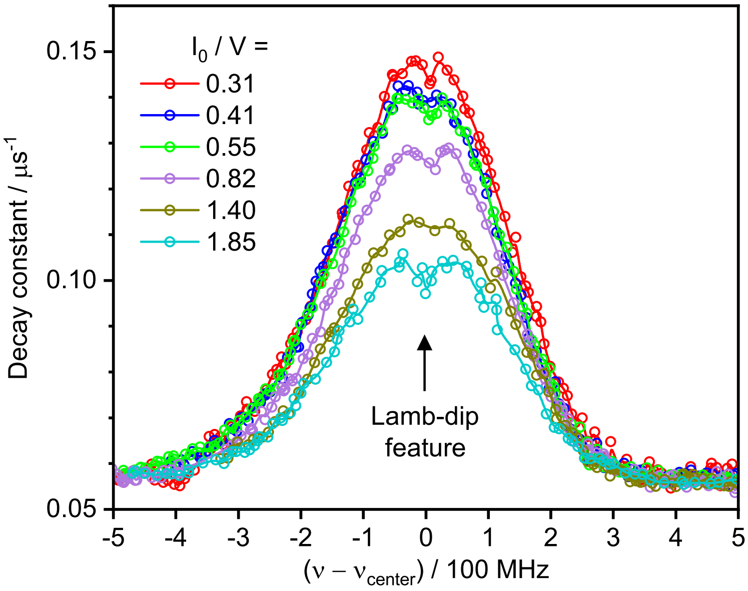

Figure S3: Lamb-dip spectra. Fitting a single exponential function to the measured saturated ringdown decays while scanning across a saturated absorption peak of CH_4 at $\tilde{\nu} = 3004.449\ \mathrm{cm}^{-1}$ (1% CH_4 in Ar, $p = 160\ \mu$bar) reveals a Lamb-dip feature at the center of the Doppler-broadened absorption line. Due to the continuous wavelength scan, the uncertainty of the wavemeter reading, and the frequency jitter resulting from the unstabilised laser source and optical cavity, the Lamb-dip feature is blurred and was difficult to reproduce. As the Doppler-free nature of CRDS did not interfere with the presented 2S1W approach, no further attempts were made to improve these measurements.

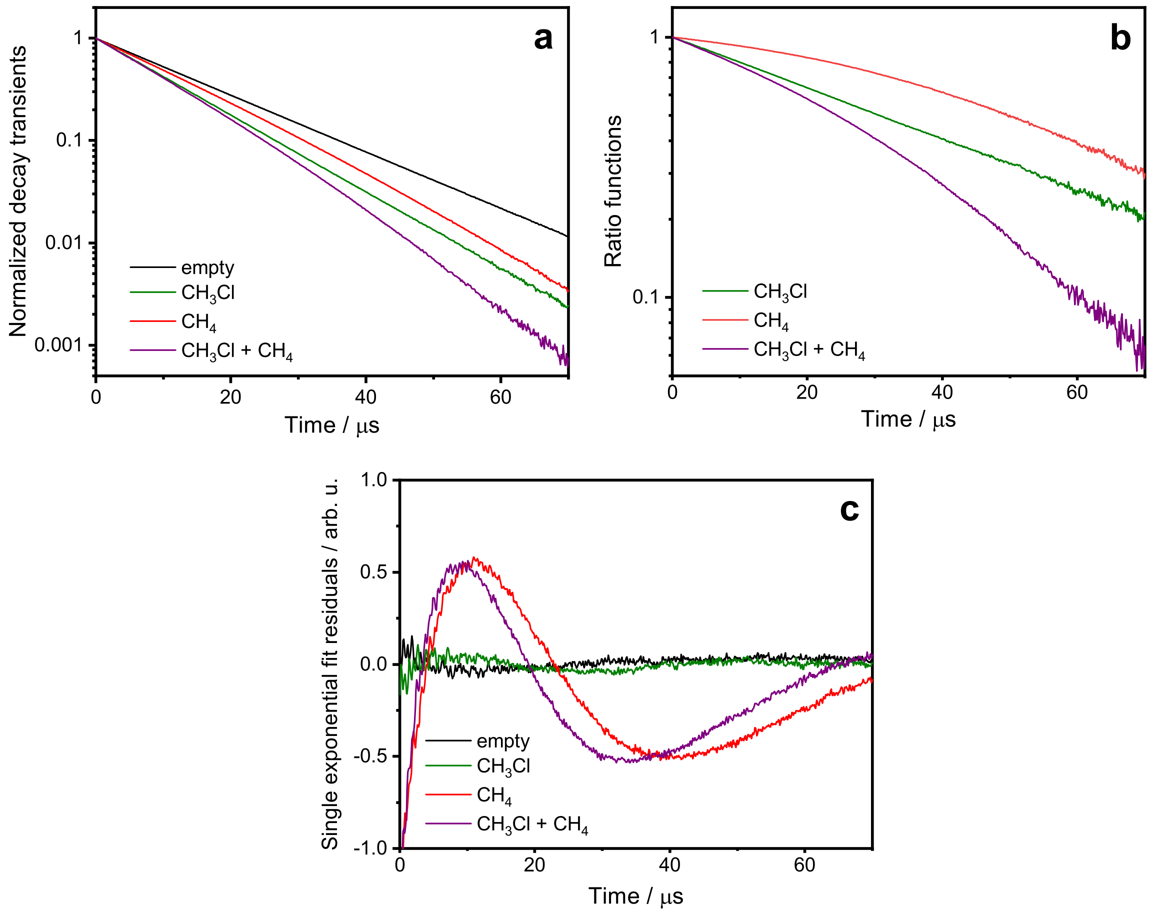

Figure S4: SCAR decay transients. Typical ringdown decays measured with the detection laser tuned to an overlapping CH_3Cl and CH_4 transition at 3057.756 cm^{-1}. Measurement parameters: total cell pressure 3.0 mbar, intracavity power 1.8 W. Panel (a): Decay transients (in a logarithmic scale) for an empty cavity (only Ar, black), a non-saturated case (only CH_3Cl added, green), a saturated case (only CH_4 added, red), and a 2S1W scenario (CH_4 and CH_3Cl added, purple). The empty cavity and CH_3Cl transients yield a linear response, while those of CH_4 and of the CH_3Cl/CH_4 mixture show a clear deviation from linearity. This can be seen more clearly in panels (b) and (c). Panel (b): Ratio functions (decays normalized with respect to the empty cavity decay) of the non-saturated case (CH_3Cl, green), the saturated case (CH_4, red) and the 2S1W scenario (CH_3Cl/CH_4 mixture, purple). Panel (c): Fit residuals of a single-exponential fit to the measured transients. Both the empty cavity and the non-saturated CH_3Cl transients show flat residuals, while those of CH_4 and CH_3Cl/CH_4 show the typical residuals (e.g., Fig 3. in Ref. [3]) of two competing decay constants. The data indicate the validity of the assumed SCAR model with only two decay constant, i.e., $\gamma_1 = \gamma_{\mathrm{empty}} + \gamma_{\mathrm{CH_3CL}}$, and $\gamma_2 = \gamma_{\mathrm{CH_4}}$.

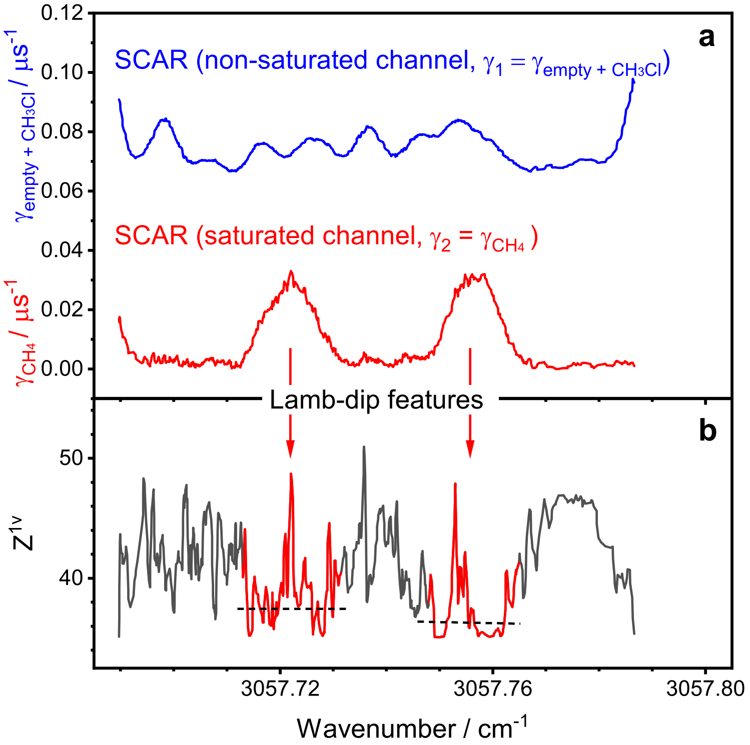

Figure S5: SCAR fit example. The plot reveals the outcome of a typical SCAR fit for an experiment with a gas mixture of 12 nbar CH_4, 30 μbar CH_3Cl, and Ar buffer gas at a total pressure of $p = 2.5$ mbar. For the fit of the SCAR transients, according to the fit model outlined in Note 2, γ_1, γ_2, S_0, $S_{\rm BG}$, and $Z^{\rm 1V}$ (constrained to $30 < Z^{\rm 1V} < 80$) have been used as the fit parameters. The resulting γ_1 and γ_2 values in panel a reflect the decoupled absorption spectra of CH_3Cl and CH_4. With $Z^{\rm 1V} = (38.4 \pm 2.8)$, a rather pronounced fluctuation has been observed for the extracted saturation parameter $Z^{\rm 1V} = C/P_{\rm s}$. This is partly due to the fact that its actual value is only important when scanning across a CH_4 absorption line. However, taking the dashed lines in panel b as the tentative baseline values for the spectral regions with significant CH_4 absorption (highlighted in red color), the marked peaks in $Z^{\rm 1V}$ (corresponding to lower $P_{\rm s}$ values) indicate a Lamb-dip effect. Note that the slightly distorted line profiles and shifted Lamb-dip positions are most likely due to inaccurate wavemeter readings for this particular experiment.

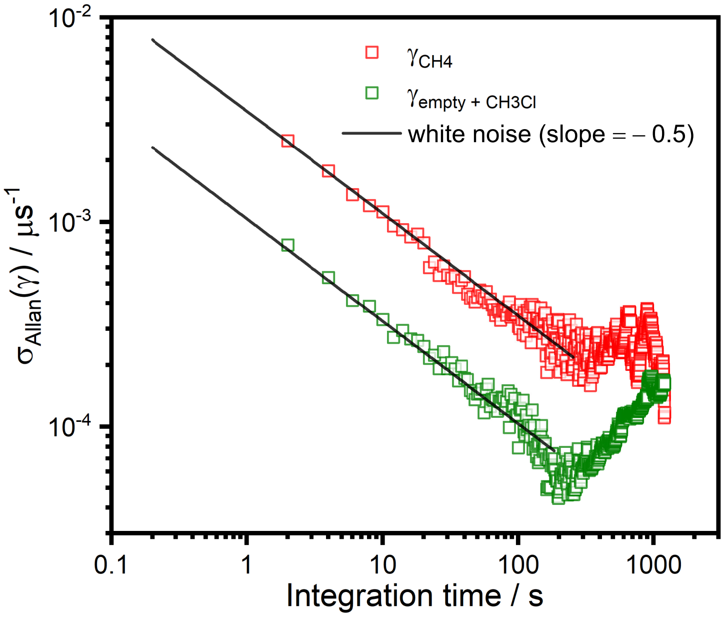

Figure S6: Allan plot. Plot of the Allan standard deviation of the extracted γ_{CH_4} (red) and $\gamma_{empty+CH_3Cl}$ (green) parameters from repeatedly measured saturated-CRD transients at the top of overlapping transitions at 3057.756 cm^{-1}. The transients were collected for a mixture of 25 μbar CH_3Cl and 8 nbar CH_4 at a total cell pressure of 2 mbar in Ar and for intracavity power of 1.2 W.

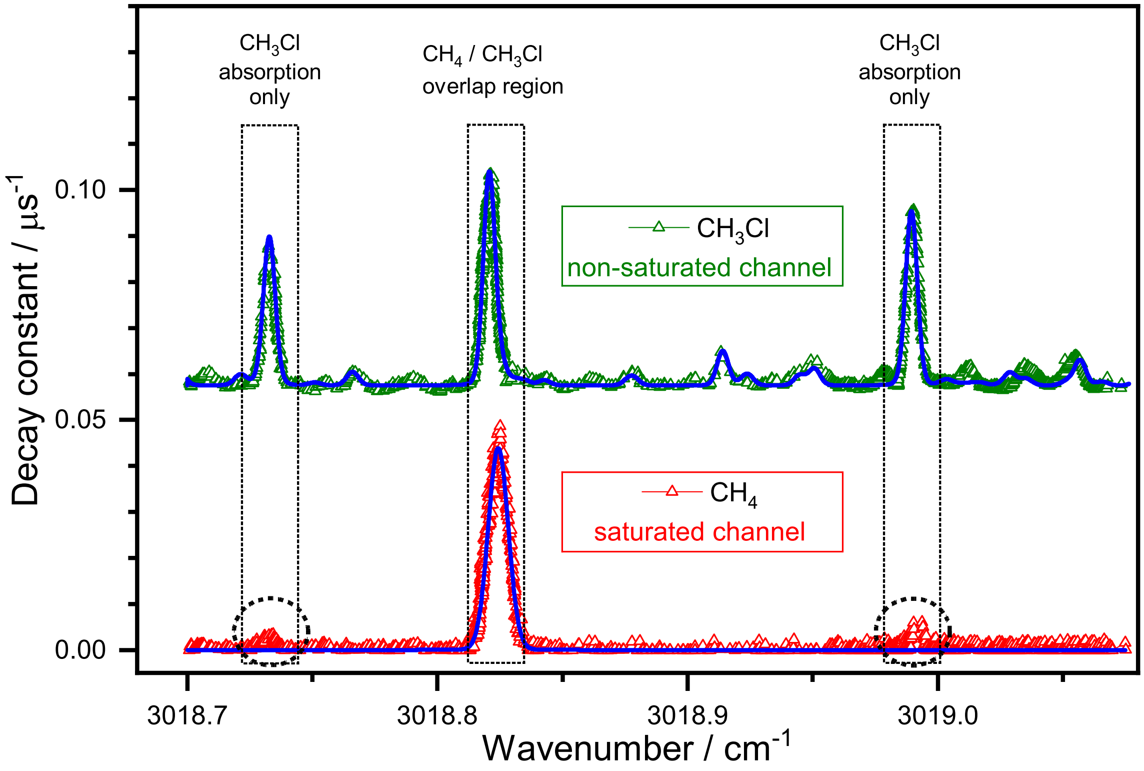

Figure S7: 2S1W linearity. Decoupled spectra from the analysis of SCAR decay transients at variable partial pressures of CH_3Cl and CH_4. Measurement parameters: Ar buffer gas, 3.0 mbar total cell pressure, 1.6 W intracavity power. Panels (a) and (b): Variable partial pressure of CH_3Cl with the partial pressure of CH_4 kept constant at 8 nbar. Panels (c) and (d): Variable partial pressure of CH_4 with the partial pressure of CH_3Cl kept constant at 30 μbar. The data reveal a very good linearity with respect to the exctracted absorptions of CH_4 and CH_3Cl, as shown in Fig. 3 (main text)

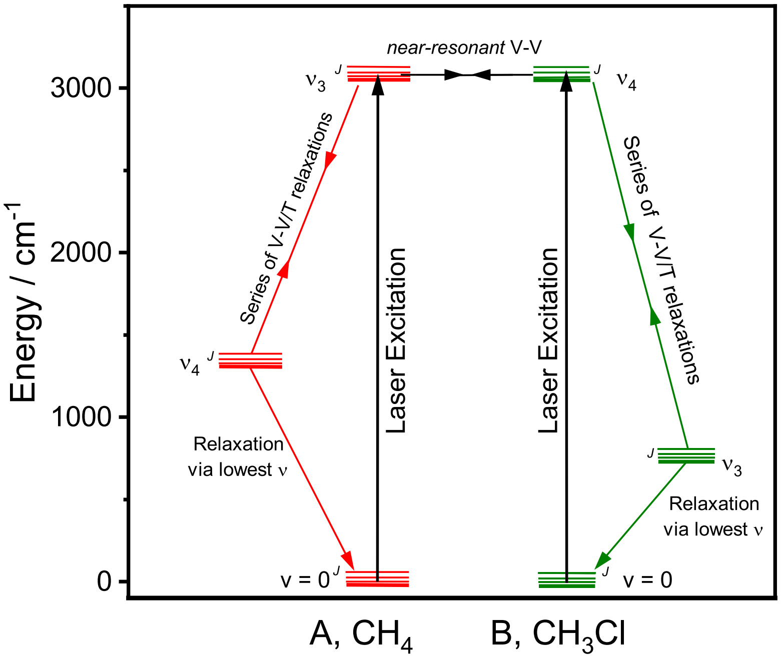

Figure S8: CH_3Cl/CH_4 energy transfer model. Schematic energy-level diagram showing vibrational energy transfer pathways for two excited polyatomic molecules A and B with the possibility of near-resonant vibration-vibration transfer. The depicted energies correspond to the model case of CH_4 and CH_3Cl. The intramolecular V-V transfer within the same molecule is often a complex and fast series of transitions ending up in the lowest vibrational mode of the molecule. Near-resonant intermolecular V-V transfer between different molecules involves quenching of vibrational energy by another relaxing molecule and is often fast compared to other relaxation pathways. Therefore, in case of efficient near-resonant V-V transfer, the molecule with the lowest vibrational state (and hence the most efficient V-T transfer to the bath gas) can serve as an efficient quencher (here, CH_3Cl) for the other molecule (here, CH_4). For high mole fraction samples (hence, a high probability for bimolecular intermolecular V-V events) such a scenario may represent a major limitation for the 2S1W approach as two species with overlapping absorption transitions and similar line strengths and relaxation constants will show a very similar degree of saturation as well.

Supplementary Tables

Table S1: V-T relaxation times. List of atmospherically relevant molecules, grouped according to their relaxation times for vibration-translation energy transfer in self-collisions at $T = 300$ K and $p = 1$ atm [1, 11].

>1000 ns	1000 - 400 ns	400 - 100 ns	100-10 ns	<10 ns
CH_4	N_2O	CH_3Cl	H_2O	CH_2ClCH_2Cl
CO_2	CS_2	C_2H_4O	C_2H_6	NH_3
COS	CF_4	C_2H_4	CH_2CHBr	CH_3CHCl
HCl	SF_6	CF_3Cl	CH_3I/Br	C_3H_7OH
HBr	CHF_3		CH_2F_2	CH_2Cl_2
CO			SO_2	C_2H_5OH
CH_3F			C_2H_2	$CHBr_2Cl$
			$CHCl_3$	$CHBrCl_2$
			$CHClF_2$	
			$CHCl_2F$	

References

[1] Lambert, J. D. *Vibrational and rotational relaxation in gases*, vol. 1 of *International series of monographs on chemistry* (Clarendon Press, Oxford, 1977).

[2] Giusfredi, G., Galli, I., Mazzotti, D., Cancio, P. & de Natale, P. Theory of saturated-absorption cavity ring-down: Radiocarbon dioxide detection, a case study. *J. Opt. Soc. Am. B* **32**, 2223-2237 (2015).

[3] Sadiek, I. & Friedrichs, G. Saturation dynamics and working limits of saturated absorption cavity ringdown spectroscopy. *Phys. Chem. Chem. Phys.* **18**, 22978–22989 (2016).

[4] Mazzotti, D., Cancio Pastor, P., De Natale, P. & Giusfredi, G. Method for measuring the concentration of trace gases by SCAR spectroscopy (2021). US Patent 10895528 B2. https://patents.google.com/patent/US10895528.

[5] Giusfredi, G. *et al.* Saturated-absorption cavity ring-down spectroscopy. *Phys. Rev. Lett.* **104**, 110801/1–4 (2010).

[6] Romanini, D., Ventrillard, I., Méjean, G., Morville, J. & Kerstel, E. Introduction to cavity enhanced absorption spectroscopy. In Gagliardi, G. & Loock, H.-P. (eds.) *Cavity-enhanced spectroscopy and sensing*, vol. 179 of *Springer Series in Optical Sciences*, chap. 11, 1–60 (Springer, 2014).

[7] Hahn, J. W., Yoo, Y. S., Lee, J. Y., Kim, J. W. & Lee, H.-W. Cavity ringdown spectroscopy with a continuous-wave laser: calculation of coupling efficiency and a new spectrometer design. *Appl. Opt.* **38**, 1859–1866 (1999).

[8] Morville, J., Romanini, D., Chenevier, M. & Kachanov, A. Effects of laser phase noise on the injection of a high-finesse cavity. *Appl. Opt.* **41**, 6980–6990 (2002).

[9] Galli, I. *et al.* Spectroscopic detection of radiocarbon dioxide at parts-per-quadrillion sensitivity. *Optica* **3**, 385–388 (2016).

[10] Gordon, I. *et al.* The HITRAN2020 molecular spectroscopic database. *J. Quant. Spectrosc. Radiat. Transfer* **277**, 107949/1–82 (2022).

[11] Lambert, J. D. Vibration-translation and vibration-rotation energy transfer in polyatomic molecules. *J. Chem. Soc. Faraday Trans. 2* **68**, 364–373 (1972).
